# Ultramicrostructural reductions in teeth: implications for dietary transition from non-avian dinosaurs to birds

**DOI:** 10.1186/s12862-020-01611-w

**Published:** 2020-04-21

**Authors:** Zhiheng Li, Chun-Chieh Wang, Min Wang, Cheng-Cheng Chiang, Yan Wang, Xiaoting Zheng, E-Wen Huang, Kiko Hsiao, Zhonghe Zhou

**Affiliations:** 1 Key Laboratory of Vertebrate Evolution and Human Origins of Chinese Academy of Sciences, Institute of Vertebrate Paleontology and Paleoanthropology, Chinese Academy of Sciences, 142 Xizhimenwai Street, Beijing, 100044 China; 2 CAS Center for Excellence in Life and Paleoenvironment, Beijing, 100044 China; 3 National Synchrotron Radiation Research Center, Hsinchu, 30076 Taiwan; 4 Institute of Geology and Paleontology, Linyi University, Linyi, 276000 Shandong China; 5 Tianyu Natural History Museum of Shandong, Pingyi, 273300 Shandong China; 6 Department of Materials Science and Engineering, National Chiao Tung University, Hsinchu, 30010 Taiwan; 7 Mr. Fossil Institute, New Taipei City, 23673 Taiwan

**Keywords:** Tooth, Avialan, Feeding ecology, Non-avian dinosaurs

## Abstract

**Background:**

Tooth morphology within theropod dinosaurs has been extensively investigated and shows high disparity throughout the Cretaceous. Changes or diversification in feeding ecology, i.e., adoption of an herbivorous diet (e.g., granivorous), is proposed as a major driver of tooth evolution in Paraves (e.g., *Microraptor*, troodontids and avialans). Here, we studied the microscopic features of paravian non-avian theropod and avialan teeth using high-spatial-resolution synchrotron transmission X-ray microscopy and scanning electron microscopy.

**Results:**

We show that avialan teeth are characterized by the presence of simple enamel structures and a lack of porous mantle dentin between the enamel and orthodentin. Reduced internal structures of teeth took place independently in Early Cretaceous birds and a *Microraptor* specimen, implying that shifts in diet in avialans from that of closely related dinosaurs may correlate with a shift in feeding ecology during the transition from non-avian dinosaurs to birds.

**Conclusion:**

Different lines of evidence all suggest a large reduction in biting force affecting the evolution of teeth in the dinosaur-bird transition. Changes in teeth microstructure and associated dietary shift may have contributed to the early evolutionary success of stemward birds in the shadow of other non-avian theropods.

## Background

Tooth reduction is one of the most conspicuous modifications characterizing the dinosaur-bird transition [1]. Morphological variation in non-avian theropod teeth is largely reflected in the different number and density of denticles on the mesial and distal carinae of teeth. This is potentially associated with a carnivorous or hyper-carnivorous feeding strategy. New microwear analyses of troodontid teeth suggests sub-optimal adaptation for typical puncture and pull feeding behavior, which could also be true for other non-avian paravian dinosaurs [2–4].

Although reductions in tooth number and distribution occurred in many stem avialan lineages toward the origin of extant birds, a great number of Mesozoic taxa retained dentition, including most enantiornithines, non-ornithothoracine avialans, and ornithuromorphs [5]. Analysis of tooth shape in terms of linear measurements in Troodontidae, Richardoestesia, Dromaeosauridae, and avialan species do not show an obvious drop in disparity throughout the Cretaceous [6]. Ecologically collapsed and deforested environments have been proposed as a major reason for the extinction of non-avian dinosaur and stem bird species, including all enantiornithines, at the end of Cretaceous [7]. The survival of avian lineages may have benefited from diversified feeding ecologies, particularly for those taxa with adoption of a granivorous diet.

Evidence of direct dietary preferences by paravian theropods, including lizard and fish pellets that are associated with a complete *Anchiornis huxleyi* skeleton, suggests that they were opportunistic feeders [8]. *Microraptor gui* reportedly preyed on mammals, birds, and even fish based on gut contents [2]. An herbivorous diet has also been proposed to be widespread among coelurosaurian dinosaurs before the origin of avialans [9]. For Mesozoic birds, the acquisition of an efficient gastric mill in several ornithuromorphs was a novel digestive feature, replacing the role of teeth in food processing. For other basal taxa that lacked a gastric mill, e.g., insectivorous and piscivorous enantiornithines [10, 11], teeth may have still played a major function in gripping prey (e.g., insects) and cutting them into small pieces. Although many functions have been inferred based on tooth morphology, there still exists substantial ambiguity in interpretations of the diet of early birds and their dinosaurian relatives [2, 3].

The adaptive changes in internal tooth structure related to a shift in feeding ecology during this critical transition are not fully understood, despite a few studies focusing on non-avian dinosaurs that are distantly related to avialans [4, 12]. Here we report the ultramicrostructural details in enantiornithines, ornithuromorphs, non-ornithothoracine avialans, and non-avian paravian theropods (microraptorines and troodontid dinosaurs) using both scanning electron microscopy (SEM) and synchrotron transmission X-ray microscopy (TXM) [13]. We found similar tooth microstructure between one *Microraptor* species and other Early Cretaceous birds. The lack of mantle dentin with interglobular porous spaces (IGS) between the enamel and orthodentin [13, 14] in avialans indicates a derived condition in Saurischian dinosaurs. In addition, the internal ultramicrostructure of teeth provides further evidence for divergent feeding ecology or food acquisition and processing in birds and closely related paravians.

## Results

### Tooth shape and enamel microstructure

**Fig. 1:**
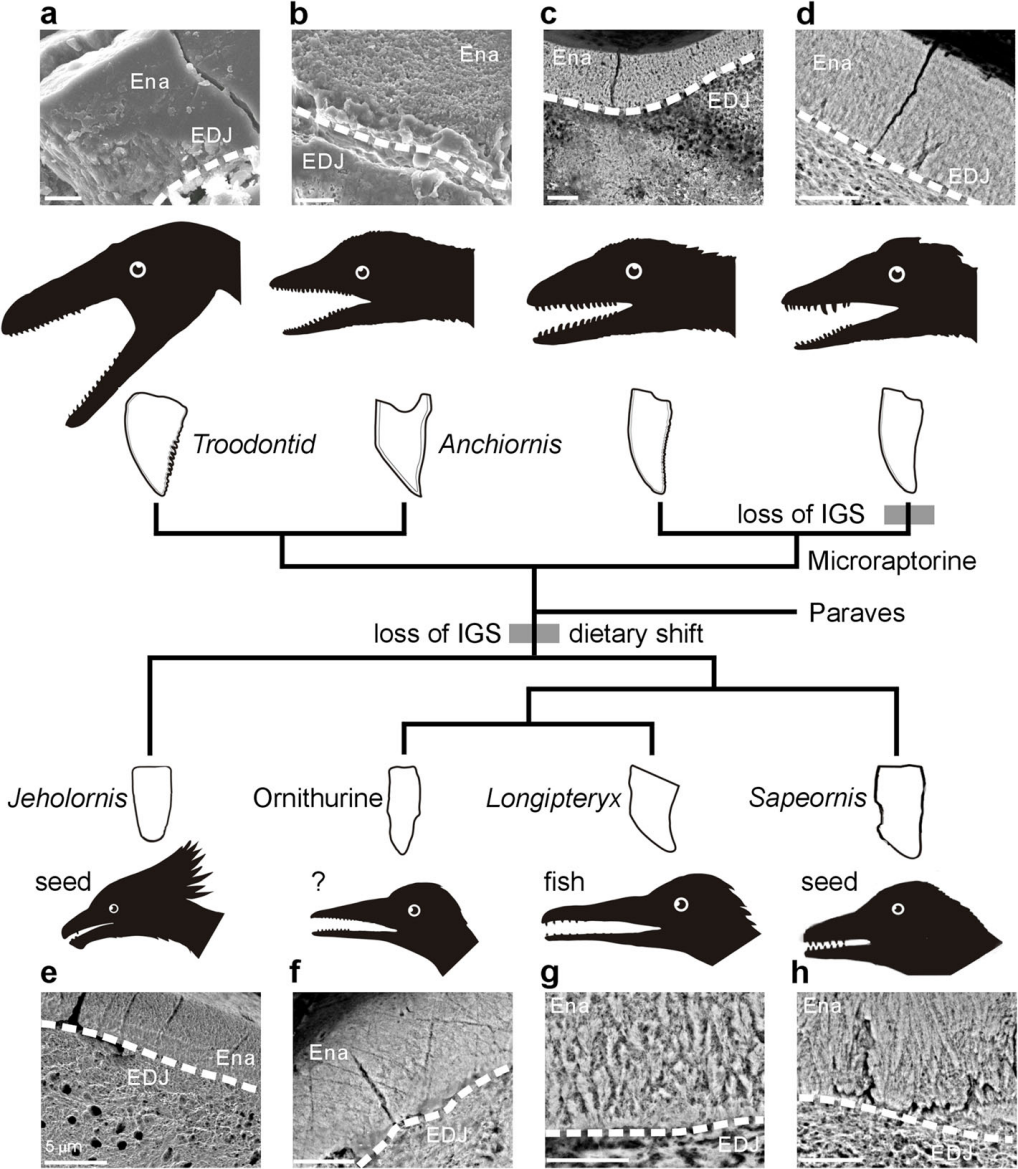
Enamel characterization and dietary evidence indicated through paravian phylogeny. SEM images showing the internal structure of sectioned teeth from Paraves with a emphasize of avialans (**a***Troodontid*, **b***Anchiornis*, **c** and **d** Microraptorines 1 and 2, **e***Jeholornis*, **f** indet. Ornithurine, **g***Longipteryx* and **h***Sapeornis.* Abbreviations: Ena, enamel; EDJ (labeled white dashed line), enamel-dentin junction. All scale bar equals to 5 μm

**Fig. 2:**
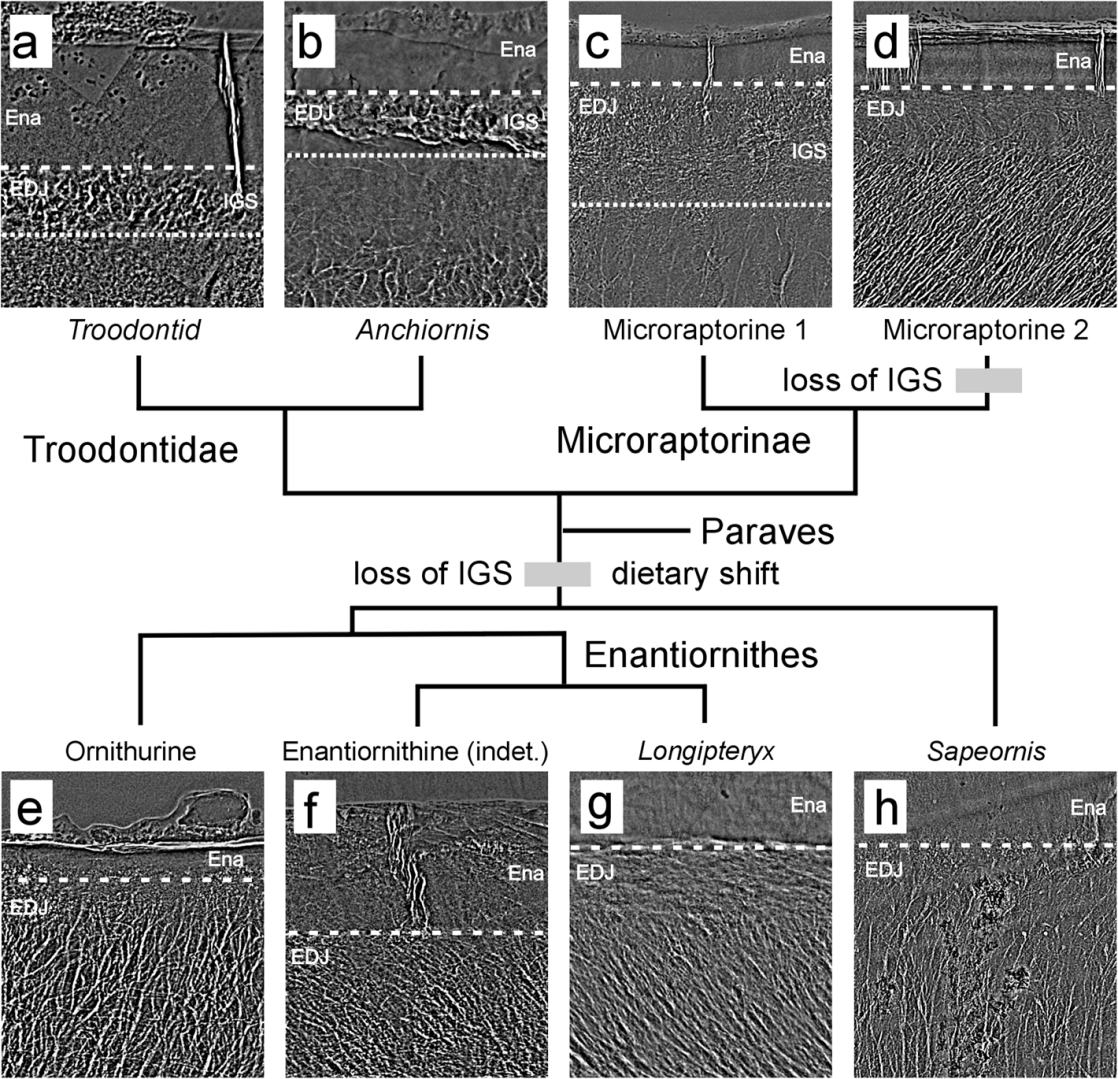
TXM image showing the internal structure of a sectioned teeth from Paraves: **a***Troodontid*, **b***Anchiornis*, **c** and **d** Microraptorine 1 and 2, **e** Enantiornithine (indet.), **f** indet. Ornithurine, **g***Longipteryx* and **h***Sapeornis*. Abbreviations: Ena, enamel; EDJ, enamel-dentin junction; IGS, interglobular porous space structure. The IGS layer is clearly present in the EDJ region of the serrated teeth of Microraptorine (**c**) and Troodontidae (**a** and **b**). The loss of IGS is labeled with a gray bar

The presence of enamel in all five bird species in this study was confirmed using SEM and TXM imaging of thin sections prepared from the tooth samples (Figs. 1, 2). Enamel thickness and ultramicroscopic structures vary among ornithuromorphs, enantiornithines, and the non-ornithothoracines *Sapeornis chaoyangensis* and *Jeholornis prima* (Fig. 1e–h), all of which suggest Mesozoic avian clade had great tooth microstructure diversity. The sampled first dentary tooth from the Enantiornithes indet. (IVPP V14606) was peg-like with a slightly caudally curved occlusal tip (Additional file 1: Figure S1). The enamel layer was characterized by a weakly developed simple, column-shaped crystalline stack (Additional file 1). The mean thickness of the enamel was approximately 6.2 μm (*n* = 8), measured from the lateral side of the base of tooth crown (Additional file 1). The ratio of the enamel thickness to the crown height (ET/CH) was about 1.8% (see Table 1), which is significantly greater than that of other fossil birds examined [15]. As the other sampled enantiornithine here, *Longipteryx chaoyangensis* only has the teeth near the rostrum (Additional file 1). The teeth are robust with a strongly caudally recurved crown (Additional file 1: Figure S2). The average thickness of enamel across the base of the crown is approximately 50 μm. The enamel is columnar with possible microunits (Fig. 1g), which have been reported in *Ichthyornis dispar* [12, 15]. The ET/CH ratio of *Longipteryx* (4%) significantly exceeds other basal avialans sampled here. Proportionally greater enamel thickness in Enantiornithes is a feature that distinguishes them from other ornithuromorph species, in addition to the recognizable gross osteological features of other skeletal elements [16].

**Table 1:** Sampled taxa and microscopic teeth character described for individual specimen

Species		Enamel feature (Terminology follows that in Hwang 2005)	Enamel Thickness (μm)	Crown height (μm)	Ratio (Enamel thickness/Crown height)	Interglobular porous mantle dentin	Inferred diet/feeding ecology	Imaging method
Indet. Ornithurine (IVPP V 14606)		Simple parallel crystallite	6.5	1119	0.58%	Absent	Herbivorous	μCT/SEM/TXM
Indet. Enantiornithine (IVPP V 16041)		Columnar enamel	6.2	346	1.8%	Absent	Insectivore	SEM/TXM
*Longipteryx chaoyangensis* (IVPP V 21702)		Columnar enamel with Crystal bundle and microunits	50.0	1260	4%	Absent	Piscivore	μCT/SEM/TXM
*Sapeornis changyangensis* (IVPP V 13759)		Columnar enamel, with partially converging crystallite	21.0	1631	1.3%	Absent	Seeds/ herbivorous	SEM/TXM
*Jeholornis prima* (IVPP V 13886)		Simple paralleled, poorly defined structure, appeared to have a few incremental lines	7.0	2956	0.2%	Absent	Seeds/ herbivorous	μCT/SEM
Microraptorine 1 and 2	Stm 5–151	Parallel crystallite enamel, with a few incremental lines close to the surface	11.1			Present	Carnivorous: lizard, bird, small animals	SEM/TXM
	Stm 5–48	Thin parallel crystallite	7.1			Absent	?	SEM/TXM
*Troodontid* sp.		Parallel crystallite	26.3			Present	General carnivorous	SEM/TXM
*Anchiornis huxleyi* (Stm 0–69)		Parallel crystallite	14.3			Present	Carnivorous: Lizard, fish,/opportunistic feeders	SEM/TXM

The dentition of *Jeholornis* was largely reduced, with only a few tiny dentary teeth and/or maxillary teeth present in known specimens [17, 18]. The bullet-shaped teeth in *Jeholornis* have a rounded crown with a cylinder-like root, and the tooth crown is nearly straight (Fig. 1e and Additional file 1: Figure S4). In the sampled maxillary teeth, the enamel layer was rather thin at about 7 μm, exhibiting a very simple paralleled crystallite arrangement and a few lines of incremental growth (Fig. 1e). The relative thickness of the enamel was 0.2% in *Jeholornis*, smaller than other birds examined here (Table 1). Reduced dentition and the adoption of a granivorous diet in *Jeholornis* may be linked. The enamel of *Sapeornis* is composed of slender columnar crystallite that partially converge (Fig. 1h and Additional file 1: Figure S3). The enamel layer in the base crown measures approximately 21 μm in thickness, which is much thicker at the tooth apex (around 49 μm) and gradually decreases toward the base (Additional file 1: Figure S9).

A cone-shaped crown was seen in the teeth of the ornithuromorph specimen. A constriction is evident between the crown and the columnar root (Fig. 1f and Additional file 1: Figure S5). The enamel thickness was about 6.5 μm on the lateral side of the crown, with an ET/CH ratio of about 0.58%, similar to the more crownward ornithurines *Hesperornis* and *Ichthyornis* [15]. The enamel is only evident within half top of the crown and composed of simple parallel crystallite. The outer enamel is more compact than the inner layer (Fig. 1f). In contrast to a previous report on indet. Avialans [19], no enamel tubules were observed in the present specimen.

### Interglobular porous mantle dentin

Interglobular dentin is found to be widely distributed in teeth of mammal, phytosaurs, non-mammalian synapsids, and ichthyosaurs, whereas the globular zones of mineralization have failed to fuse into a homogeneous mass within mature dentin [20–24]. Located between the dentin and enamel, the porous interglobular space identified here is inside mantle dentin of saurischian dinosaurs and *Alligator*, mostly occurring within the tooth crown. This particular less-dense porous tissue plays a key role in the redistribution of stress [13]. The IGS layer was absent from the microraptorine tooth (Microraptorine 2 in Fig. 2) and all the avialan teeth sampled in the present study (Fig. 2a–d). By contrast, the presence of IGS in another microraptorine specimen (Microraptorine 1 in Fig. 2) and in troodontids was confirmed in the mantle dentin region (Fig. 2a–c). Moreover, the dentinal tubules of the serrated microraptorine tooth and troodontid teeth extended into the IGS layer (Fig. 2). In contrast, in all the avialan teeth studied here the dentinal tubules end slightly below the EDJ. A similar condition was found in the unserrated microraptorine tooth (Microraptorine 1 in Fig. 2). We propose that the loss of the IGS layer is a derived feature that evolved independently in Microraptorinae and in the most recent common ancestor of Avialae (Figs. 1 and 2). The enamel cracks in both enantiornithines and the unserrated microraptorine teeth never extended into the bulk dentin (Fig. 2d, f). By contrast, in the serrated microraptorine and troodontid teeth, the extension of cracks was arrested by the IGS layer beneath the enamel (Fig. 2a, c). Prior analysis of IGS has shown the crack-arresting properties of this layer, created by mismatched elastic modulus between enamel and dentin [20]. This observation further suggests different mechanical properties and teeth usage that was optimal for different feeding behaviors within Paraves [25].

## Discussion

Tooth reduction across the dinosaur-bird transition has been proposed to result from a feeding ecology that shifted from mainly carnivorous or hypercarnivorous to a more herbivorous or omnivorous diet [3]. This dietary shift/diversification is supported by both direct and indirect evidence, including food residue in the gut, the presence of gizzard stones (gastroliths), and the appearance of a muscular crop and other related digestive features [10, 26]. The role of teeth in feeding has been generally overlooked in early birds and is rarely considered to play a major role.

Variations in gross morphology in avialan teeth were immediately recognized in the numerous new bird fossils discovered from the Early Cretaceous Jehol Biota (Fig. 1 and Additional file 1: Figures S1–S5). These include the most commonly found peg-shaped teeth in many enantiornithines and ornithuromorphs, e.g., *Cathayornis*, *Eoenantiornis*, and *Yanornis*. Taxa with unusual teeth forms have also been reported, including large-sized robust teeth in *Sulcavis geeorum* [11], strongly curved teeth in *Longipteryx*, dome-shaped teeth in *Pengornis* [16], and very tiny teeth in *Hongshanornis* and *Eogranivora* [27, 28]. The shift away from obligate carnivorous feeding in Cretaceous stem birds facilitate the changes in feeding apparatus, that allowed the raw morphology of teeth to diversify into different shapes [2, 18]. Teeth number also differs significantly among the aforementioned taxa, ranging from only several in *Jeholornis*, to over 20 in *Yanornis* [29]. In addition to number and external morphology, internal micro-structures characterizing avialan teeth were also revealed in the present paper.

Enantiornithes are distinct in having relatively thick enamel compared to other Mesozoic stem birds. This unique characteristic may support previously made proposals regarding the durophagous dietary preferences (eating of tough materials) of several taxa [11, 16]. For instance, *Sulcavis* has been observed to have robust dentition with deep grooves on exterior tooth surfaces [11]. The crystal shapes that form the enamel structure in these birds also potentially play a functional role in feeding. For instance, in non-avian dinosaurs, paralleled crystal shaped enamel has been proposed to be superior to columnar shaped enamel in resisting wear and abrasion [12]. The presence of microunits enamel and piscivorous diet in *Longipteryx* could be correlated as also seen from piscivorous ichthyosaurs [12]. The lack of complexed enamel in most Cretaceous birds suggests their substantially weakened role in resisting wear during food processing. The simplified and thin enamel layer may also be related to a fast incubation time and a lack of adequate time for mineral deposition [30, 31]. In comparison to the commonly reported paralleled enamel found in dromaeosaurids [32], even enantiornithines with relatively thick enamel show a simplified crystal structure without a clearly layered structure. The absence of a paralleled structure indicates enamel reduction compared with other Maniraptora [32].

Given that most non-avian theropods and other saurischian dinosaurs possess interglobular porous mantle dentin, the loss of this particular tissue is a key feature in bird teeth [13, 33]. A previous study showed its absence in ornithischian dinosaurs (e.g., Hadrosauridae and Marginocephalia) [14, 34]. However, the enamel spindles identified in ornithischians are absent from the EDJ region in avian species, distinguishing these two clades. In addition, all Mesozoic birds examined to date lack the wavy enamel found in ornithischian dinosaurs [13, 14, 19].

In addition to tooth number reduction, the loss of interglobular porous mantle dentin that occurred in the non-avian dinosaur-to-bird transition is associated with changes in feeding ecology and the functional role of teeth during food acquisition. Interestingly, the lack of interglobular porous mantle dentin found in avialan species has also been observed in a newly discovered Microraptorian tooth, which lacks serrations (Fig. 1d). Convergent evolution in feeding could play a major role in shaping dental traits in these closely related bird lineages [9]. This hypothesis is supported by the derived hyolingual feeding adaptation that co-occurred in the two lineages [35]. The simple columnar crystallite enamel and thin parallel enamel both suggest that biting or raptorial feeding ecology is unlikely for most stem Mesozoic birds and even for small paravian theropods.

New data have also revealed diversity in bird teeth regarding both gross and microscopic structures. For example, tooth denticles were proposed as an indicator of a puncture-and-pull function in carnivorous theropods [4]. Reduced or weakly developed denticles have also been found in a few paravian theropods (e.g., *Microraptor* and *Anchiornis*), and this structure is absent from all avialans. Reductions of enamel thickness and the structure of schmelzmuster in ornithurine and other early bird teeth may be further associated with the appearance of the gastric mill used for grinding. Changing to an herbivorous diet could relax the selection pressure to maintain the tooth shape and denticles used for a carnivorous diet, giving rise to increased raw teeth variations. For these reasons, we infer that collecting and/or holding food items rather than processing is the major function of such bird teeth.

Small-bodied theropods diversified quickly during the Late Jurassic period. In the face of intensified competition from carnivorous theropods and pterosaurs, changes of feeding ecology to a more herbivorous diet could be one of the major selective factors ruling on the evolution of Cretaceous stem birds, as exemplified by tooth changes in both exterior morphology and internal microstructures. In addition to feeding adaptation, a recently hypothesized developmental process (i.e., incubation duration) may also have constrained tooth growth [30]. Fast embryonic growth and a shortened incubation time may have restricted tooth development compared with non-avian theropods with a longer period of incubation [31].

We propose that a large reduction in biting force due to an innovative dietary shift acted as a major factor affecting the evolution of teeth in the dinosaur-bird transition. This was characterized by the loss of interglobular porous mantle dentin and simplification of the enamel structure. Direct evidence from one specimen of *Microraptor* feeding on enantiornithines suggests that biting remained vital for foraging behavior in some Microraptorine taxa. Moreover, we identified a new Microraptorian specimen that resembled avialan species, which featured unserrated teeth across both the upper and lower jaws, and more importantly also the similar internal ultramicrostructures. Increased cranial kinesis and reduced robustness occurred concurrent with modifications in tooth structure in Paraves [36]. These lines of evidence all suggest further reductions in birds in the biting force inherited from their common paravian ancestor. Changes in tooth morphology suggest that dietary shifts may have acted as an adaptive response that allow and increase the diversification of early birds in the face of competition with other carnivorous non-avian theropods.

## Methods

### Taxon sampling

We sampled five representatives of avialan teeth (Table 1) to investigate microstructural diversity in comparison with their closest outgroup taxa, Dromaeosauridae (Microraptorine taxa) and troodontids using both scanning electron microscopy (SEM) and/or synchrotron transmission X-ray microscopy (TXM). The high-resolution images show delicate structural differences in the teeth of these taxa and provide new evidence regarding their dietary preferences.

Our sampling covers major avialan lineages, including stem ornithuromorphs, enantiornithine, basal avialans, and also the paravian dinosaurs (Table 1, Additional file 1: Figures S1-S9), majority of which are from the Jehol specimens deposited at the Institute of Vertebrate Paleontology and Paleoanthropology (IVPP), Tianyu Natural History Museum of Shandong (STM), and Paleo Wonders Museum (PWM). We sampled one dentary tooth from a small-sized enantiornithine (IVPP V16041) and a larger *Longipteryx* (IVPP V21702) dentary tooth, one isolated Troodontid tooth specimen (PWM 5400400036) and one *Anchiornis* dentary tooth (STM 0–69), as well as half of an isolated tooth from one Microraptorine (1) specimen (STM 5–48) and a complete dentary tooth from another Microraptorine (2) specimen (STM 5–151). Other avian sample numbers are as follows: Ornithuromorph sp. (IVPP V14606), *Jeholornis prima* (IVPP V13886) and *Sapeornis chanyangensis* (IVPP V13759).

### TXM preparation

In preparing thin sections for observation with transmission X-ray microscopy (TXM), we sliced the epoxy-embedded fossil teeth to a thickness of 50–100 μm using a Leica SP1600 Saw Microtome, and sequentially hand-polished the sliced teeth to a thickness of 20–30 μm using silicon carbide 120, 240, 500, 800, 1200, 2500, and 4000 polishing paper to minimize the scratches on the specimen surfaces. No other complicated preparation processes were needed for observation. The original internal structure inside the tooth section was not destroyed during the preparation process. The TXM at beamline BL01B1 of the Taiwan Light Source provided two-dimensional radiography and three-dimensional tomography with an approximately 60-nm spatial resolution. A superconducting wavelength shifter source of BL01B1 beamline provided a photon flux of 4 × 10^11^ photons s^− 1^ (0.1% bw)^− 1^ in the energy range 5–20 keV. A double crystal monochromator exploiting a pair of Ge (111) crystals selected x-rays with 8–11 keV of energy. A Fresnel zone-plate was used as an objective lens to magnify x-ray images. Conjugated with a 20× downstream scintillator-based optical magnification, the microscope provided a total magnification of 880×. The field of view for each image was 15 × 15 μm^2^, however, a millimeter scale field of view can also be provided by stitching images of the specimens obtained from sequential positions.

### SEM section preparation

Polished sections of bird teeth were etched with 0.1 mol/L phosphoric acid for about 90 s, treated in an ultrasonic bath, and air-dried. Air-dried specimens were examined and acquired on a Zeiss Gemini SEM 500 at the Instrument Analysis Center of Xi’an Jiaotong University and the Chinese Academy of Geological Sciences (FEI Quanta 450 FEG) in Beijing. Backscattered-Electron (BSE) Imaging were taken at voltages of 1–20 kV.

## Supplementary information

**Additional file 1: Figure S1** The rostrum of indet. Enantiornitine, IVPP V 16041 (left: Photo, and right: Computed Laminography image). White arrow indicates the dentary taken from this small-sized enantiornithine specimen. The conical-shaped tooth is small and only slightly curved caudally. The cervix of the tooth is rather wide with a narrow crown. **Figure S2** The rostrum of a referred specimen of *Longipteryx chaoyangensis* (IVPP V 21702). White arrow indicates the dentary tooth fell off from the rostrum. The tooth is relatively large and strongly curved caudally with a wide cervix (see the inserted image showing the 3D surface rendering from micro-CT scan of the tooth taken). **Figure S3** Skull of a referred specimen of *Sapeornis chaoyangensis* (IVPP V13759). The isolated maxillae tooth was sampled (White arrow pointed). The tooth is rather large and columnar in shape. No significant expansion is present on the tooth cervix. The tip of tooth become slightly pointed. **Figure S4** Close up image of the tooth sampled from a referred specimen of *Jeholornis prima* (IVPP V 13886). Cross-section of the tooth is very rounded from the root to the crown and only gradual decrease of diameter is visible in the 3D surface rendering model of the micro-CT scan (right inset). **Figure S5** The tooth sampled from a new ornithuromorph specimen (IVPP V 14606). The tooth shape is distinct with regards to its expanded columnar shaped root with a constriction of the crown (see the inserted image of the 3D surface rendering model from the micro-CT scan). **Figure S6** Skull of a new Microraptorine specimen 2 (STM 5–151). The isolated tooth with a sickle-like shape was sampled for sectioning. **Figure S7** Skull of a new Microraptorine specimen 1 (STM 5–48). One isolated half dentary tooth was sampled from this specimen. **Figure S8**. Disarticulated skull of a new specimen of *Anchiornis huxleyi* (STM 0–69). White arrow indicates the dentary tooth sampled. **Figure S9** SEM imaging to show the enamel structure and measurements taken from each tooth.

## Data Availability

All relevant data are available from the authors and/or are included within the manuscript and Additional file.

## Abbreviations

EDJ: Enamel-dentin junction
IGS: Interglobular porous spaces
IVPP: Institute of Vertebrate Paleontology and Paleoanthropology
PWM: Paleo Wonders Museum
STM: Tianyu Natural History Museum of Shandong
TXM: Transmission X-ray microscopy

## References

[1] Meredith RW, Zhang G, Gilbert MTP, Jarvis ED (2014). Springer MS: evidence for a single loss of mineralized teeth in the common avian ancestor. Science.

[2] O’Connor JK (2019). The trophic habits of early birds. Palaeogeogr Palaeoclimatol Palaeoecol.

[3] Zanno LE, Makovicky PJ (2011). Herbivorous ecomorphology and specialization patterns in theropod dinosaur evolution. Proc Natl Acad Sci.

[4] Torices A, Wilkinson R, Arbour VM, Ruiz-Omeñaca JI, Currie PJ (2018). Puncture-and-pull biomechanics in the teeth of predatory Coelurosaurian dinosaurs. Curr Biol.

[5] Zhou Z, Li FZZ (2009). A new lower cretaceous bird from China and tooth reduction in early avian evolution. Proceedings of the Royal Society B*:* biological sciences.

[6] Larson DW, Brown CM, Evans DC (2016). Dental disparity and ecological stability in bird-like dinosaurs prior to the end-cretaceous mass extinction. Curr Biol.

[7] Field DJ, Bercovici A, Berv JS, Dunn R, Fastovsky DE, Lyson TR (2018). Early evolution of modern birds structured by global Forest collapse at the end-cretaceous mass extinction. Curr Biol.

[8] Zheng X, Wang X, Sullivan C, Zhang X, Zhang F, Wang Y (2018). Exceptional dinosaur fossils reveal early origin of avian-style digestion. Sci Rep.

[9] Zanno LE, Makovicky PJ (2010). Herbivorous ecomorphology and specialization patterns in theropod dinosaur evolution. Proc Natl Acad Sci.

[10] Wang M, Zhou Z, Sullivan C (2016). A fish-eating Enantiornithine bird from the early cretaceous of China provides evidence of modern avian digestive features. Curr Biol.

[11] O’Connor JK, Zhang Y, Chiappe LM, Meng Q, Quanguo L, Di L (2013). A new Enantiornithine from the Yixian formation with the first recognized avian enamel specialization. J Vertebr Paleontol.

[12] Sander MP (1999). The microstructure of reptilian tooth enamel: terminology, function, and phylogeny. Münchn geowiss Abh.

[13] Wang C-C, Song Y-F, Song S-R, Ji Q, Chiang C-C, Meng Q (2015). Evolution and function of dinosaur teeth at Ultramicrostructural level revealed using synchrotron transmission X-ray microscopy. Sci Rep.

[14] Brink KS, Reisz RR, LeBlanc ARH, Chang RS, Lee YC, Chiang CC (2015). Developmental and evolutionary novelty in the serrated teeth of theropod dinosaurs. Sci Rep.

[15] Dumont M, Tafforeau P, Bertin T, Bhullar B-A, Field D, Schulp A (2016). Synchrotron imaging of dentition provides insights into the biology of Hesperornis and ichthyornis, the “last” toothed birds. BMC Evol Biol.

[16] Zhou Z, Clarke J, Zhang F (2008). Insight into diversity, body size and morphological evolution from the largest early cretaceous enantiornithine bird. J Anat.

[17] Zheng X, Wang X, O’Connor J, Zhou Z (2012). Insight into the early evolution of the avian sternum from juvenile enantiornithines. Nat Commun.

[18] O’Connor JK, Chiappe LM (2011). A revision of enantiornithine (Aves: Ornithothoraces) skull morphology. J Syst Palaeontol.

[19] Hwang SH (2011). The evolution of dinosaur tooth enamel microstructure. Biol Rev.

[20] Bechtle S, Fett T, Rizzi G, Habelitz S, Klocke A, Schneider GA (2010). Crack arrest within teeth at the dentinoenamel junction caused by elastic modulus mismatch. Biomaterials.

[21] LeBlanc Aaron RH, Brink Kirstin S, Whitney Megan R, Abdala F, Reisz Robert R (2018). Dental ontogeny in extinct synapsids reveals a complex evolutionary history of the mammalian tooth attachment system. Proc R Soc B Biol Sci.

[22] Maxwell EE, Caldwell MW, Lamoureux DO (2012). Tooth histology, attachment, and replacement in the Ichthyopterygia reviewed in an evolutionary context. Paläontol Z.

[23] Tjäderhane L, Carrilho MR, Breschi L, Tay FR, Pashley DH (2009). Dentin basic structure and composition—an overview. Endod Top.

[24] Nanci A (2017). Ten Cate’s Oral histology-E-book: development, structure, and function: Elsevier health sciences.

[25] Xu X, Zhou Z, Wang X (2000). The smallest known non-avian theropod dinosaur. Nature.

[26] Zheng X, Martin LD, Zhou Z, Burnham DA, Zhang F, Miao D (2011). Fossil evidence of avian crops from the early cretaceous of China. Proc Natl Acad Sci U S A.

[27] Chiappe LM, Zhao B, O’Connor JK, Chunling G, Wang X, Habib M (2014). A new specimen of the early cretaceous bird Hongshanornis longicresta: insights into the aerodynamics and diet of a basal ornithuromorph. PeerJ.

[28] Zheng X, O’Connor JK, Wang X, Wang Y, Zhou Z (2018). Reinterpretation of a previously described Jehol bird clarifies early trophic evolution in the Ornithuromorpha. Proc R Soc B Biol Sci.

[29] Zhou Z, Clarke J, Zhang F, Wings O (2004). Gastroliths in Yanornis: an indication of the earliest radical diet-switching and gizzard plasticity in the lineage leading to living birds?. Naturwissenschaften.

[30] Yang T-R, Sander PM (2018). The origin of the bird's beak: new insights from dinosaur incubation periods. Biol Lett.

[31] Erickson GM, Zelenitsky DK, Kay DI, Norell MA (2017). Dinosaur incubation periods directly determined from growth-line counts in embryonic teeth show reptilian-grade development. Proc Natl Acad Sci.

[32] Button K, You H, Kirkland JI, Zanno L (2017). Incremental growth of therizinosaurian dental tissues: implications for dietary transitions in Theropoda. PeerJ.

[33] Feng R, Maley JM, Schatte G, Hoffmeyer RE, Brink KS, Ellis T (2016). Chemical and structural information from the enamel of a Troodon tooth leading to an understanding of diet and environment. Appl Spectrosc.

[34] Brink KS, Chen Y-C, Wu Y-N, Liu W-M, Shieh D-B, Huang TD (2016). Dietary adaptions in the ultrastructure of dinosaur dentine. J R Soc Interface.

[35] Li Z, Zhou Z, Clarke JA (2018). Convergent evolution of a mobile bony tongue in flighted dinosaurs and pterosaurs. PLoS One.

[36] Hu H, Sansalone G, Wroe S, McDonald PG, O’Connor JK, Li Z (2019). Evolution of the vomer and its implications for cranial kinesis in Paraves. Proc Natl Acad Sci.

