# Supplementary material for: Ultramicrostructural reductions in teeth: implications for dietary transition from non-avian dinosaurs to birds
Source: BMC Evol Biol. 2020 Apr 21;20:46. doi: 10.1186/s12862-020-01611-w (PMC7171806; doi:10.1186/s12862-020-01611-w)
Supplement: Supplementary file 1 — Additional file 1: Figure S1 The rostrum of indet. Enantiornitine, IVPP V 16041 (left: Photo, and right: Computed Laminography image). White arrow indicates the dentary taken from this small-sized enantiornithine specimen. The conical-shaped tooth is small and only slightly curved caudally. The cervix of the tooth is rather wide with a narrow crown. Figure S2 The rostrum of a referred specimen of Longipteryx chaoyangensis (IVPP V 21702). White arrow indicates the dentary tooth fell off from the rostrum. The tooth is relatively large and strongly curved caudally with a wide cervix (see the inserted image showing the 3D surface rendering from micro-CT scan of the tooth taken). Figure S3 Skull of a referred specimen of Sapeornis chaoyangensis (IVPP V13759). The isolated maxillae tooth was sampled (White arrow pointed). The tooth is rather large and columnar in shape. No significant expansion is present on the tooth cervix. The tip of tooth become slightly pointed. Figure S4 Close up image of the tooth sampled from a referred specimen of Jeholornis prima (IVPP V 13886). Cross-section of the tooth is very rounded from the root to the crown and only gradual decrease of diameter is visible in the 3D surface rendering model of the micro-CT scan (right inset). Figure S5 The tooth sampled from a new ornithuromorph specimen (IVPP V 14606). The tooth shape is distinct with regards to its expanded columnar shaped root with a constriction of the crown (see the inserted image of the 3D surface rendering model from the micro-CT scan). Figure S6 Skull of a new Microraptorine specimen 2 (STM 5–151). The isolated tooth with a sickle-like shape was sampled for sectioning. Figure S7 Skull of a new Microraptorine specimen 1 (STM 5–48). One isolated half dentary tooth was sampled from this specimen. Figure S8. Disarticulated skull of a new specimen of Anchiornis huxleyi (STM 0–69). White arrow indicates the dentary tooth sampled. Figure S9 SEM imaging to show the enamel structure and meas [file 12862_2020_1611_MOESM1_ESM.docx]

**Additional file**

**Ultramicrostructural Reduction of Teeth: Implications for Dietary Transition from Non-avian Dinosaurs to Birds**

Z. Li, C.C. Wang, M.Wang *et al*


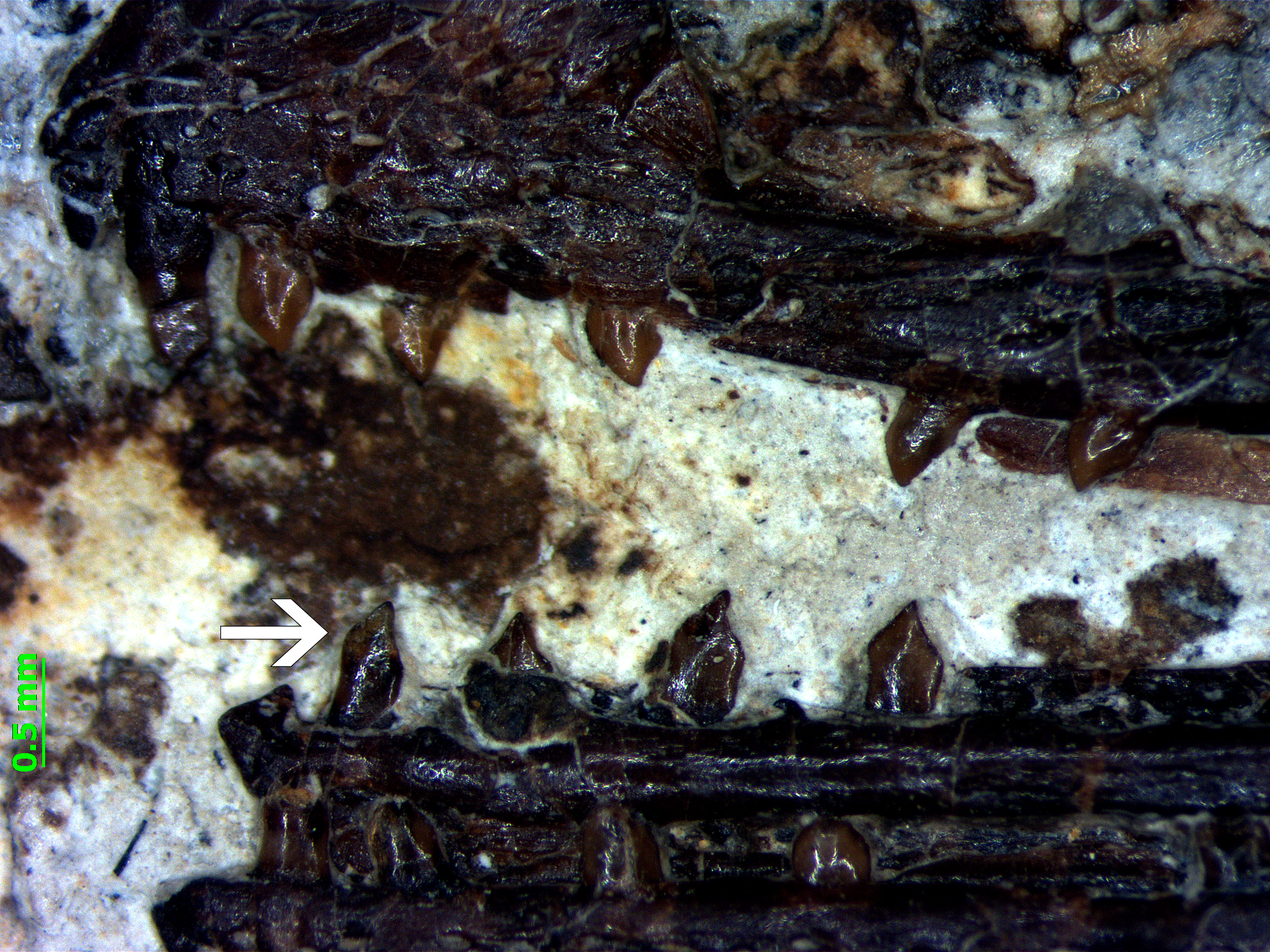

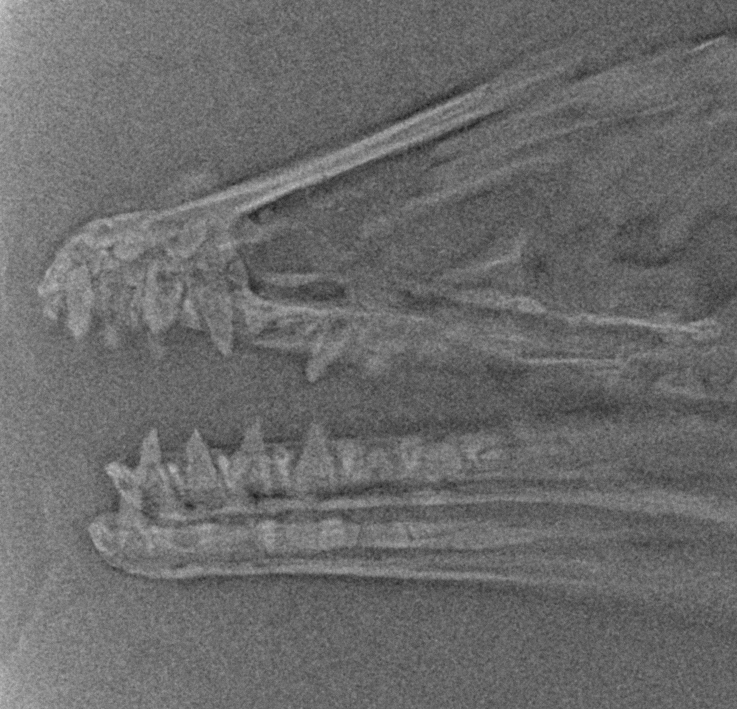


**Figure S1.** The rostrum of indet. enantiornitine, IVPP V 16041 (left: Photo, and right: Computed Laminography image). White arrow indicates the dentary taken from this small-sized enantiornithine specimen. The conical-shaped tooth is small and only slightly curved caudally. The cervix of the tooth is rather wide with a narrow crown.


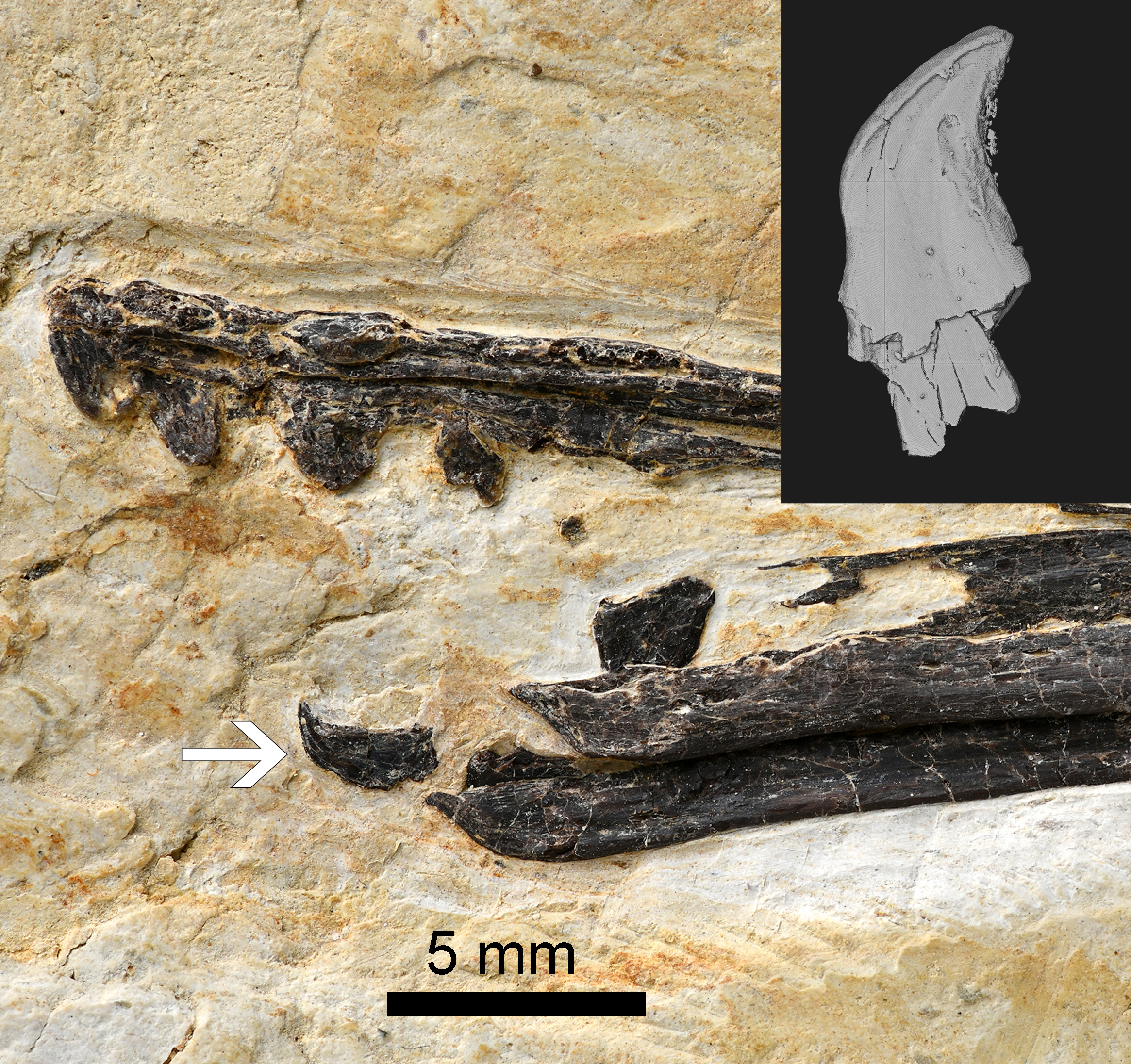


**Figure S2.** The rostrum of a referred specimen of *Longipteryx chaoyangensis* (IVPP V21702). White arrow indicates the dentary tooth fell off from the rostrum. The tooth is relatively large and strongly curved caudally with a wide cervix (see the inserted image showing the 3D surface rendering from micro-CT scan of the tooth taken).


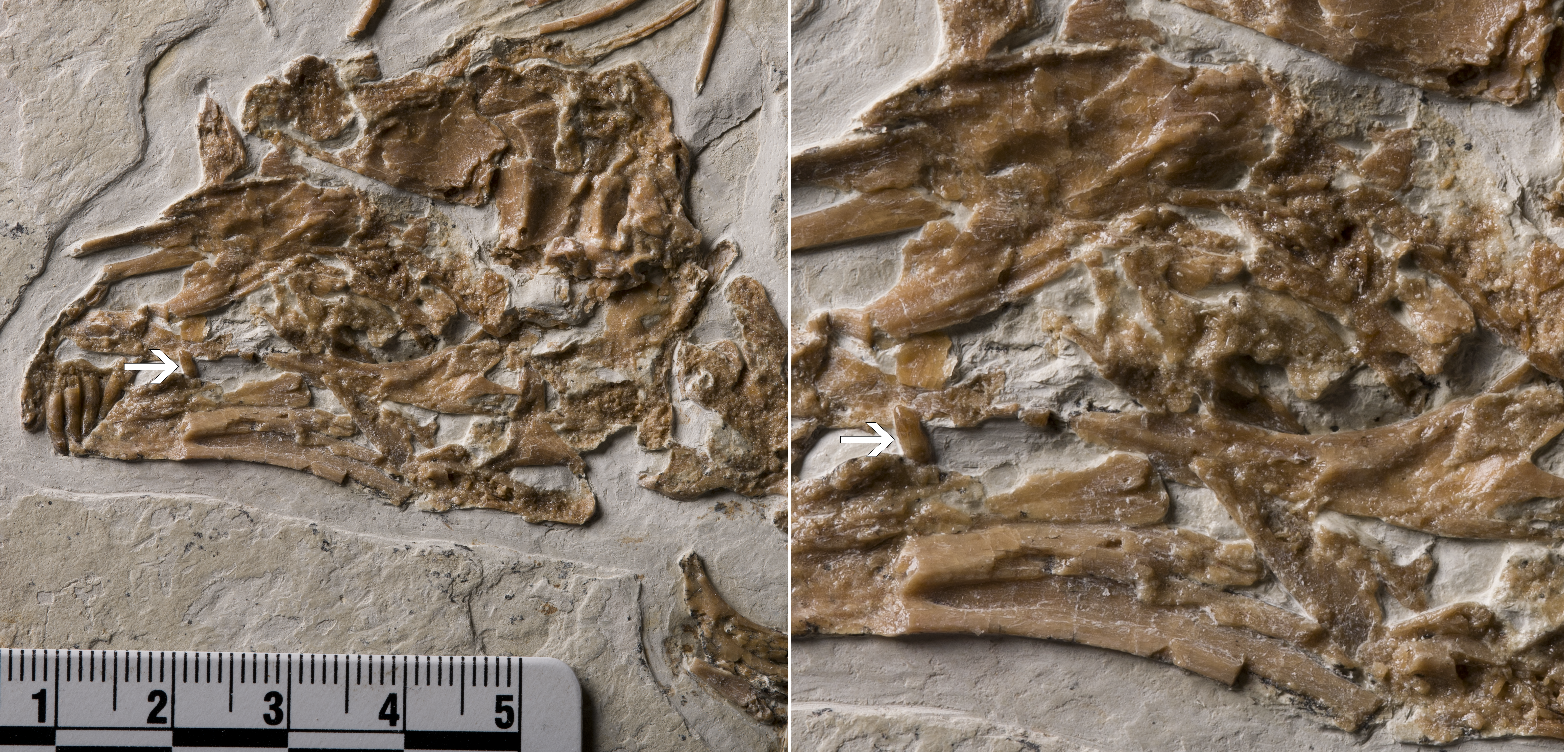


**Figure S3.** Skull of a referred specimen of *Sapeornis chaoyangensis* (IVPP V13759). The isolated maxillae tooth was sampled (White arrow pointed). The tooth is rather large and columnar in shape. No significant expansion is present on the tooth cervix. The tip of tooth become slightly pointed.


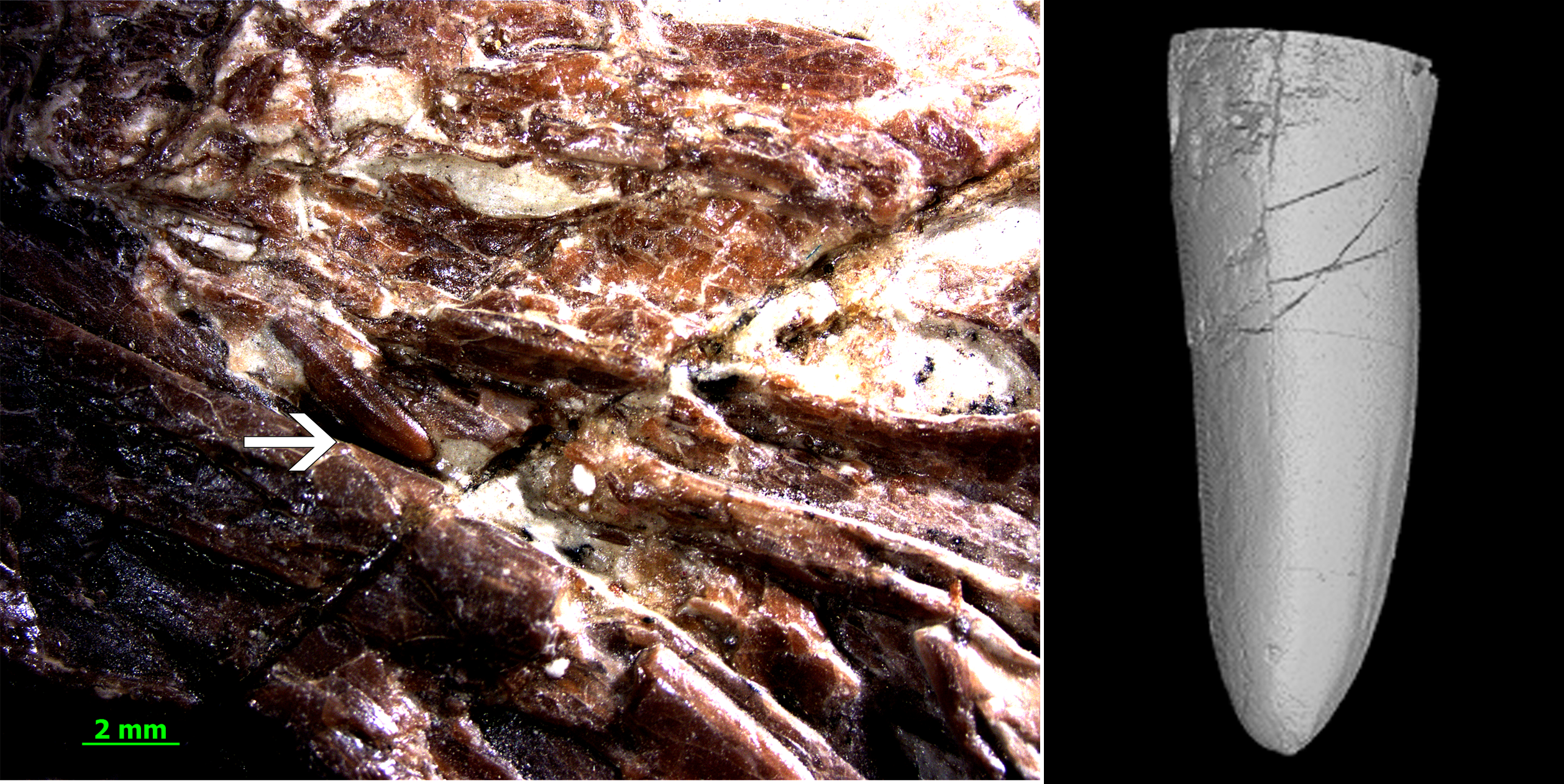


**Figure S4.** Close up image of the tooth sampled from a referred specimen of *Jeholornis prima* (IVPP V13886). Cross-section of the tooth is very rounded from the root to the crown and only gradual decrease of diameter is visible in the 3D surface rendering model of the micro-CT scan (right inset).


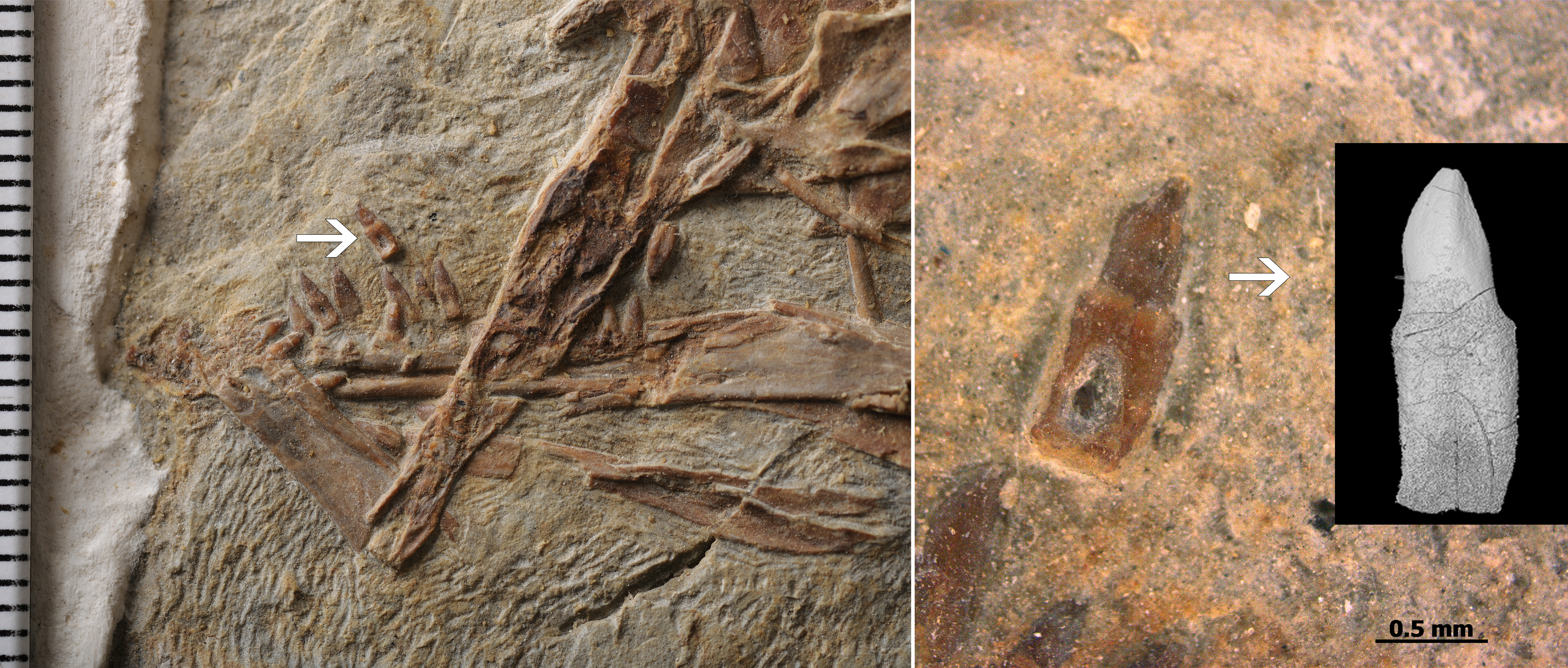


**Figure S5.** The tooth sampled from a new ornithuromorph specimen (IVPP V14606). The tooth shape is distinct with regards to its expanded columnar shaped root with a constriction of the crown (see the inserted image of the 3D surface rendering model from the micro-CT scan).


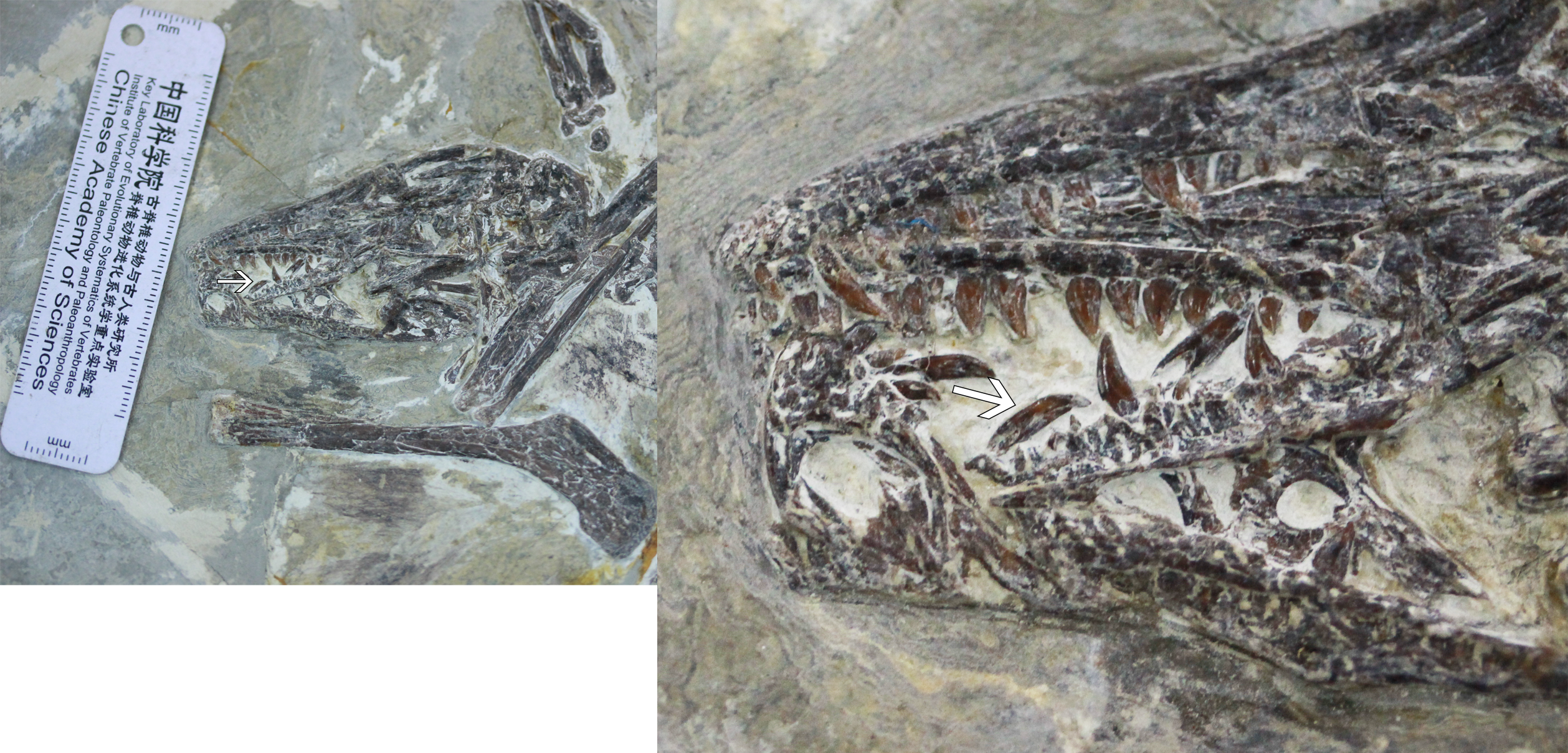


**Figure S6**. Skull of a new Microraptorine specimen 2 (STM 5-151). The isolated tooth with a sickle-like shape was sampled for sectioning.


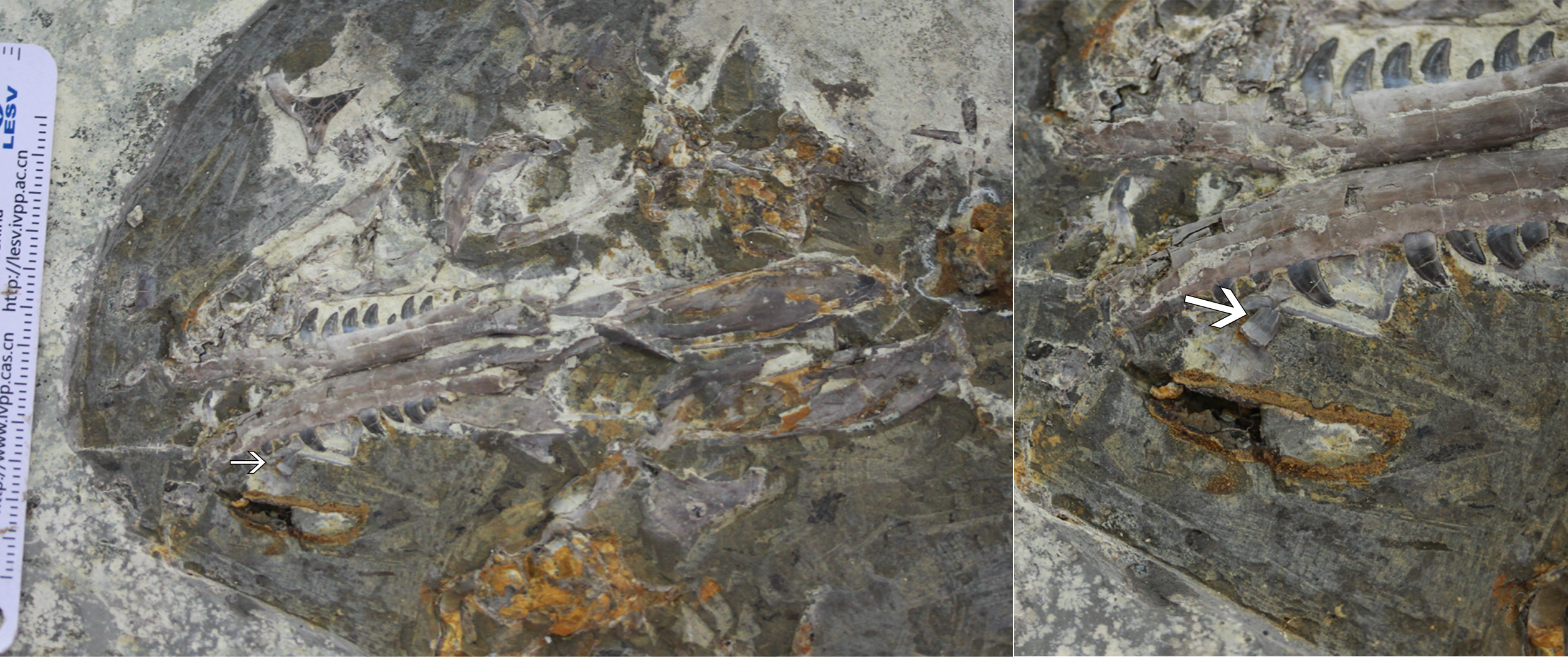


**Figure S**7. Skull of a new Microraptorine specimen 1 (STM 5-48). One isolated half dentary tooth was sampled from this specimen.


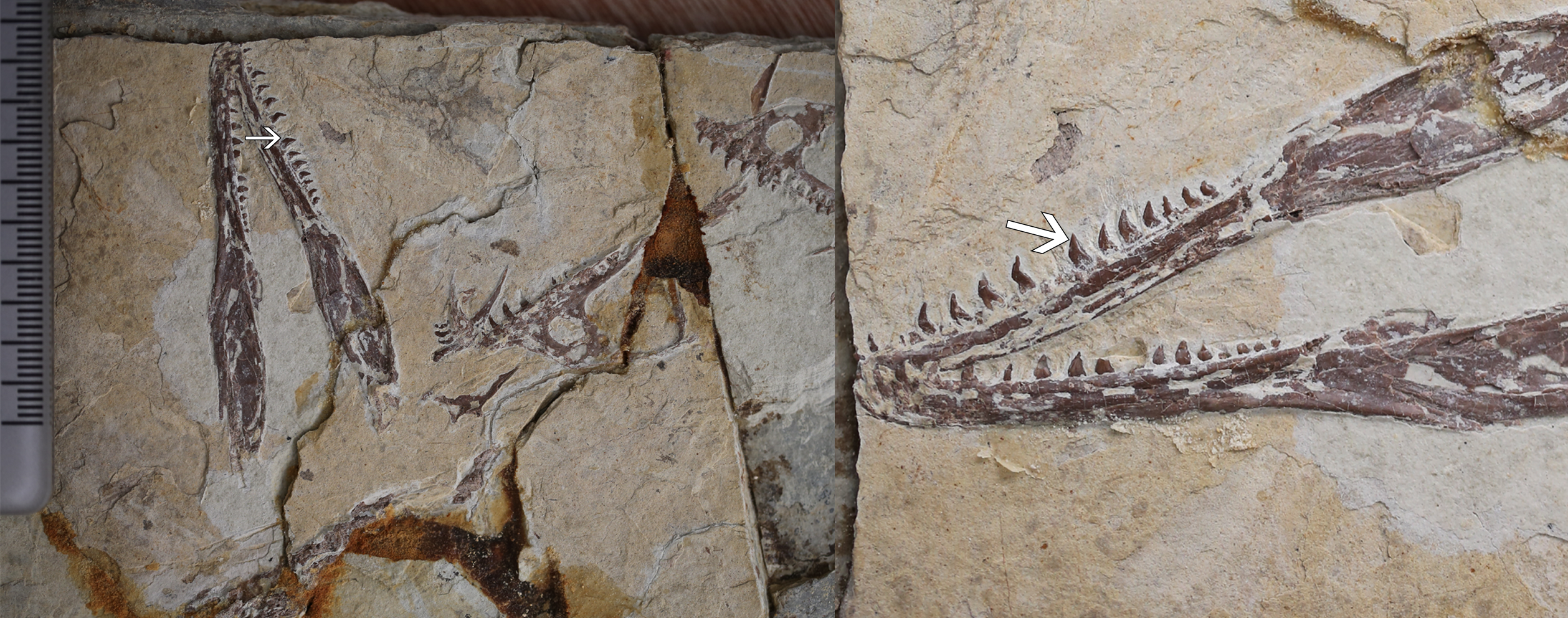


**Figure S8**. Disarticulated skull of a new specimen of *Anchiornis huxleyi* (STM 0-69). White arrow indicates the dentary tooth sampled.

**Figure S9****.** SEM imaging to show the enamel structure and measurements taken from each tooth.


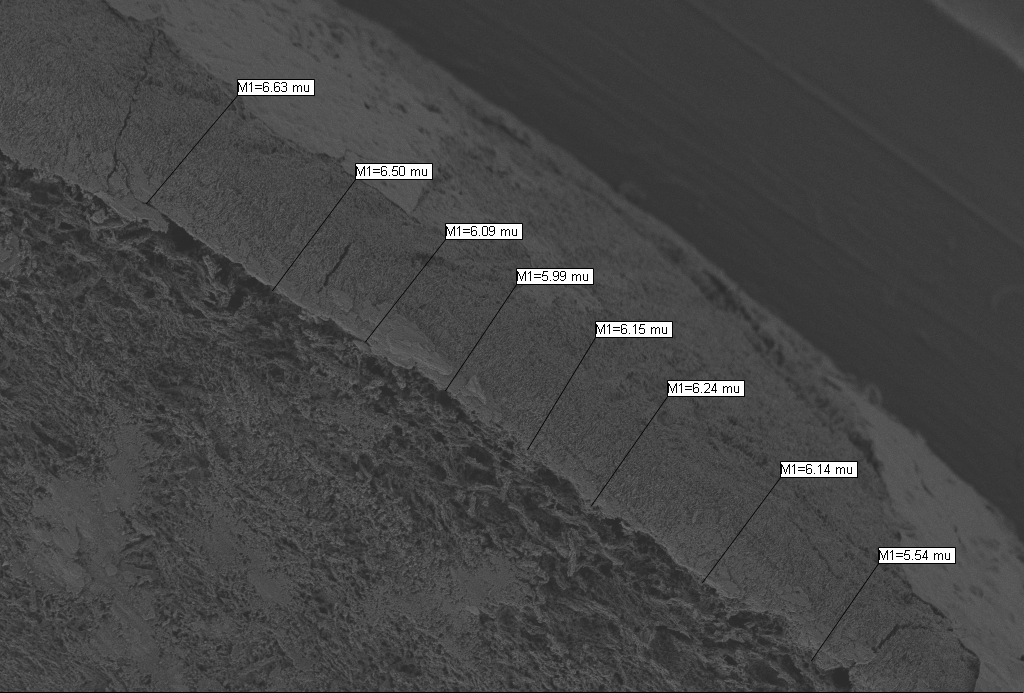

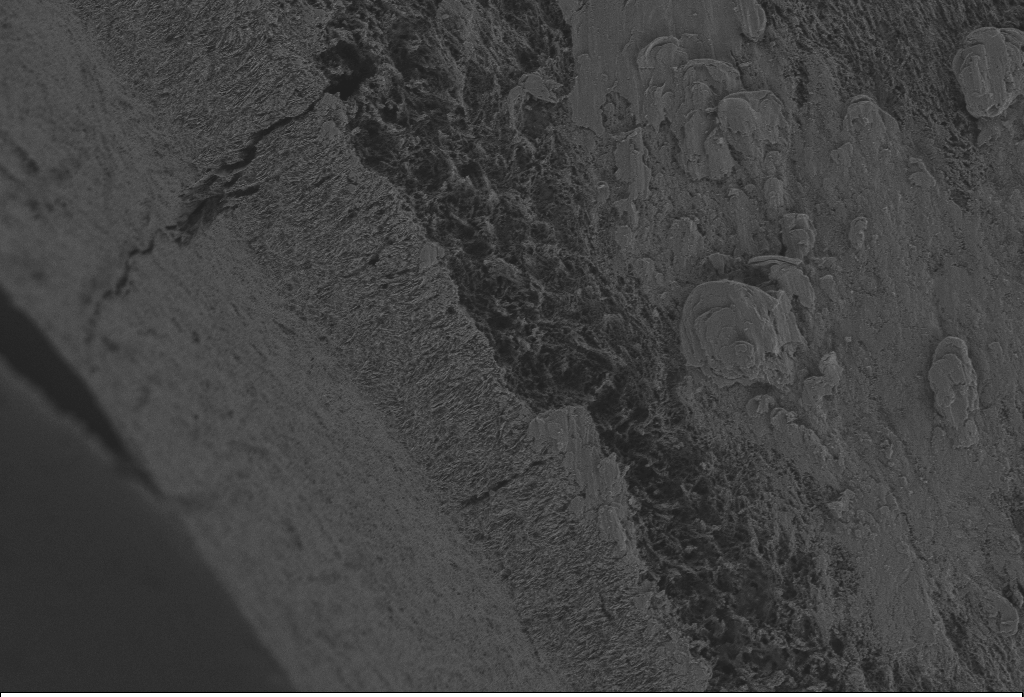


Indet. Enantiornithine (IVPP V 16041)


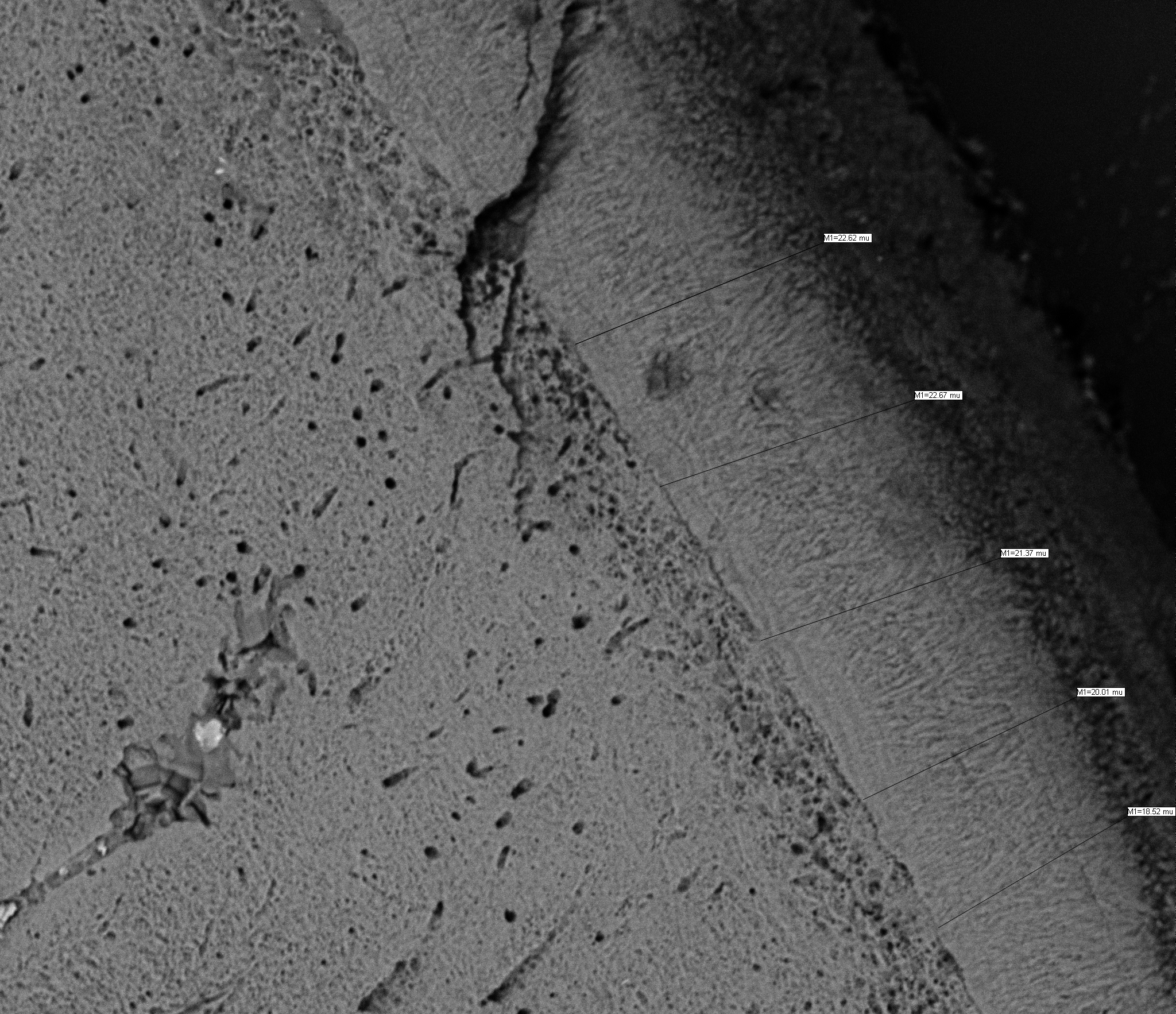

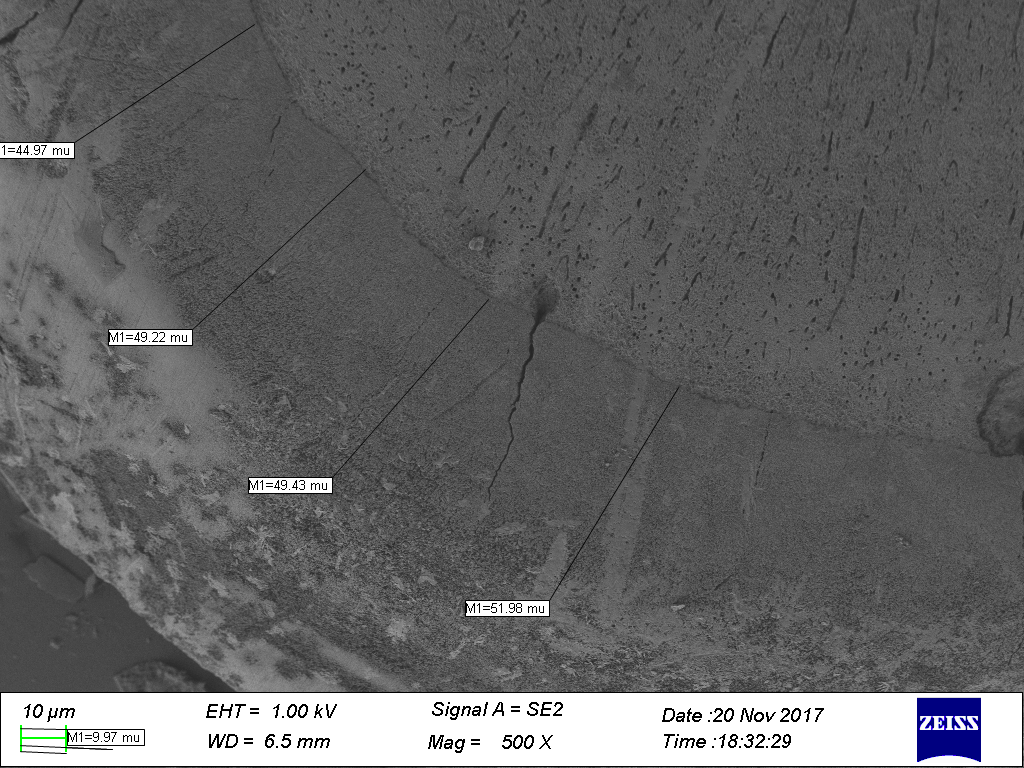


*Sapeornis* *chaoyangensis* (IVPP V 13759) Left: middle of the crown; right: close to the apex.


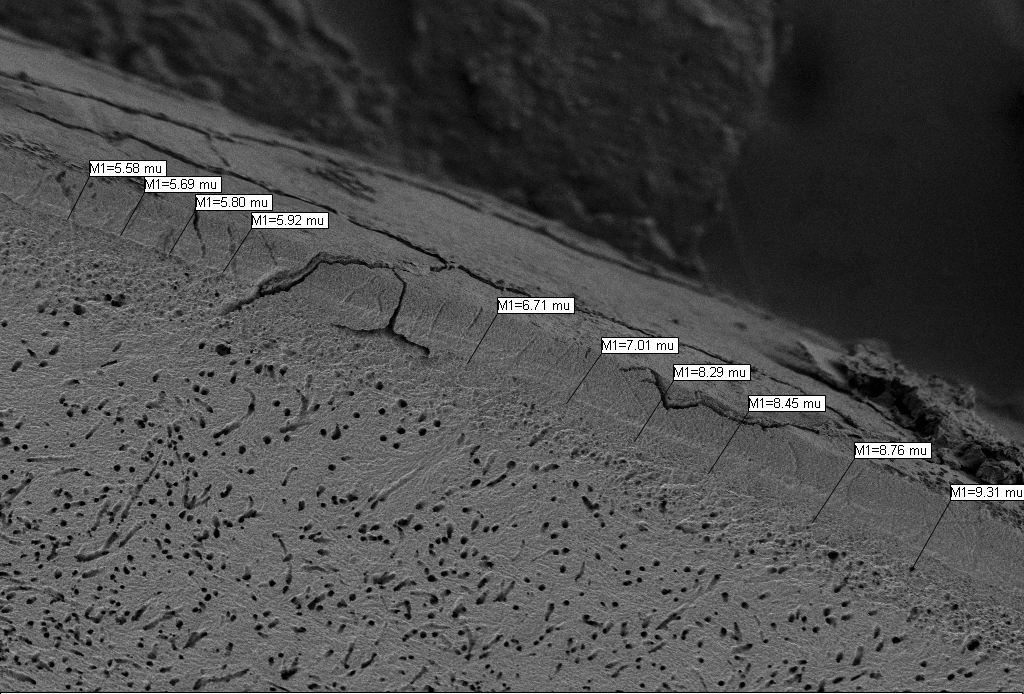


*Jeholornis* *prima* (IVPP V 13886)


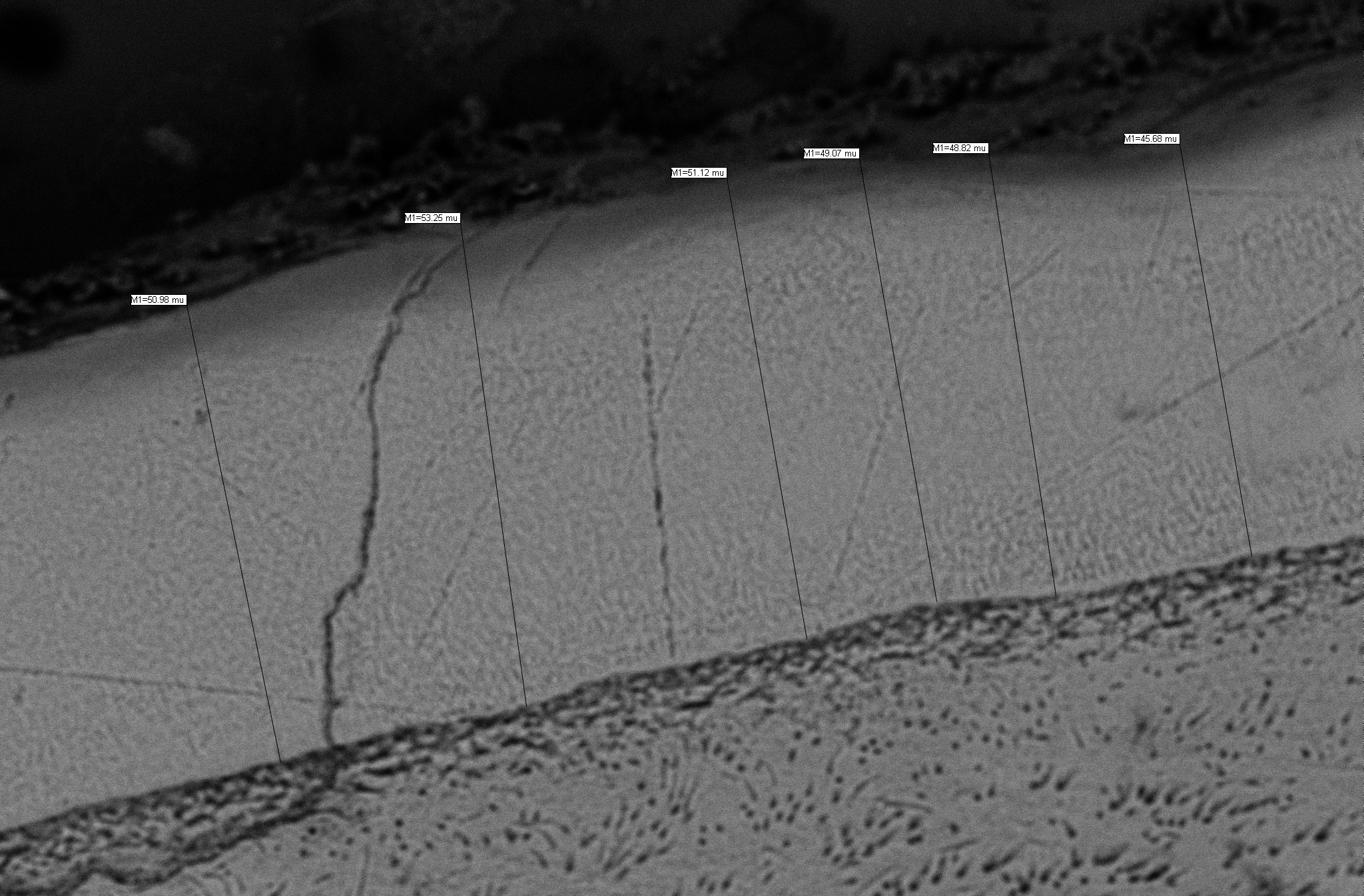


*Longipteryx* *chaoyangensis* (IVPP V 21702)


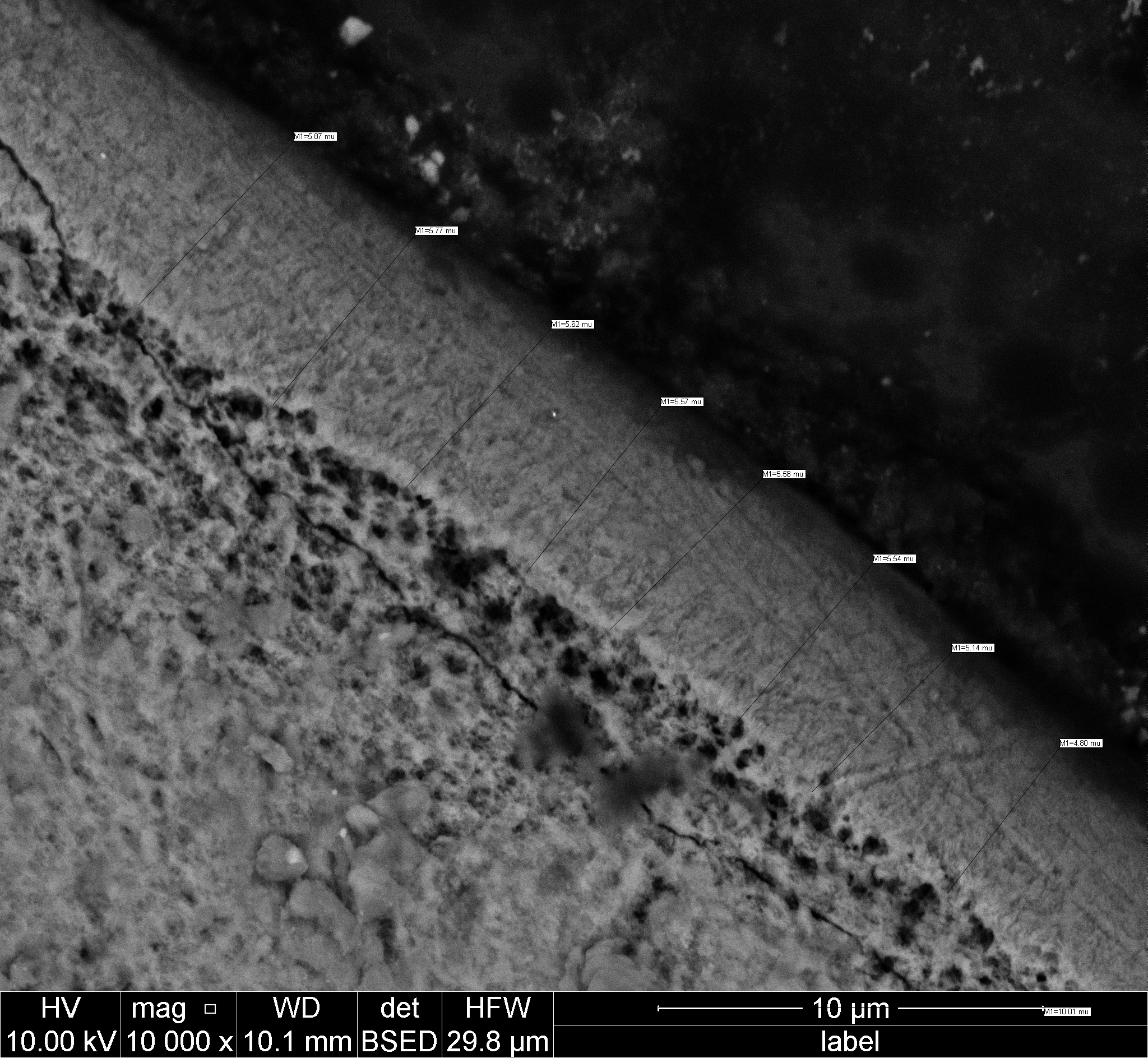


Indet. Ornithuromorph specimen (IVPP V 14606)


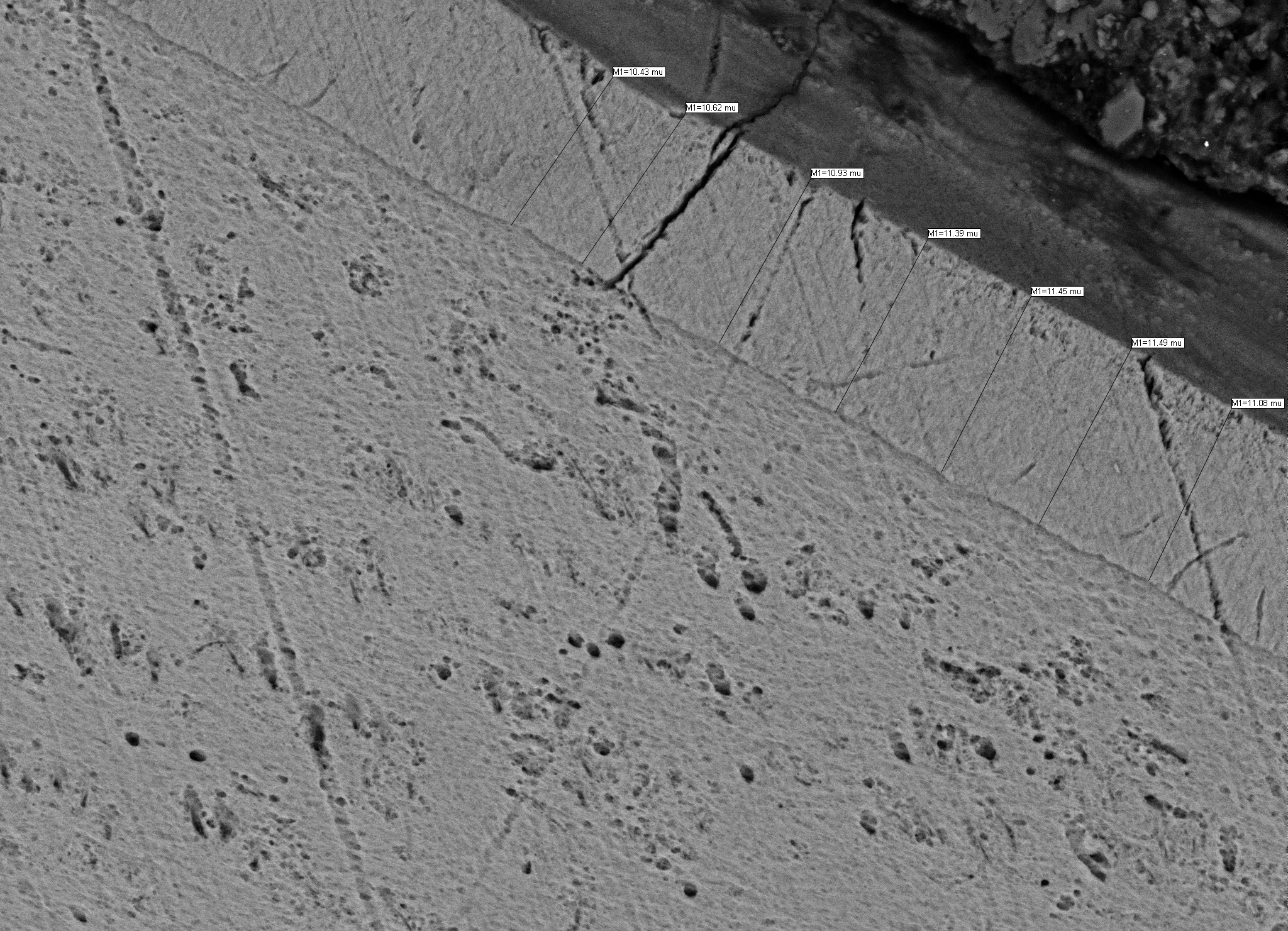


Indet. Microraptorine 2 (STM 5-151)


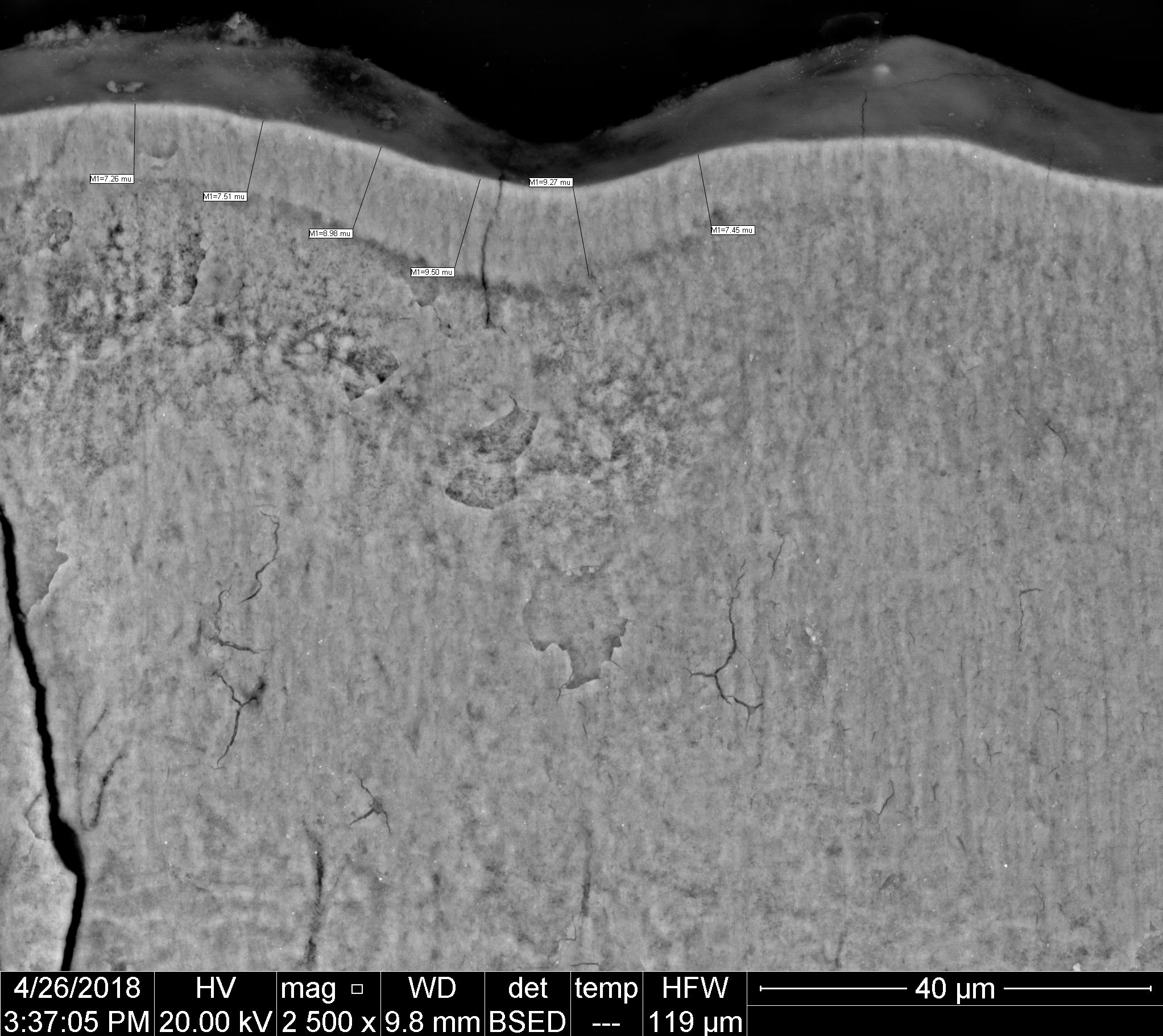


Indet. Microraptorine 1 (STM 5-48)


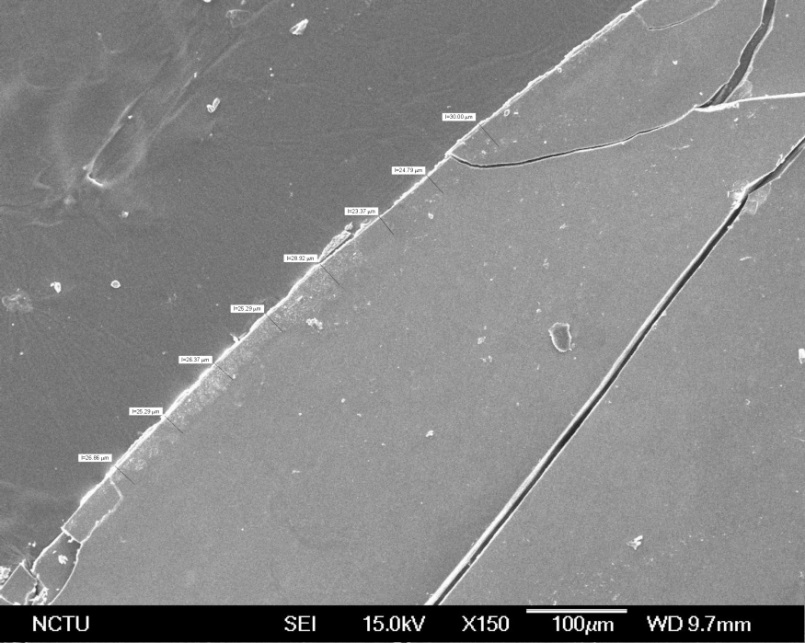


*Troodontid* sp. (PWM 5400400036)





*Anchiornis huxleyi* (STM 0-69)
